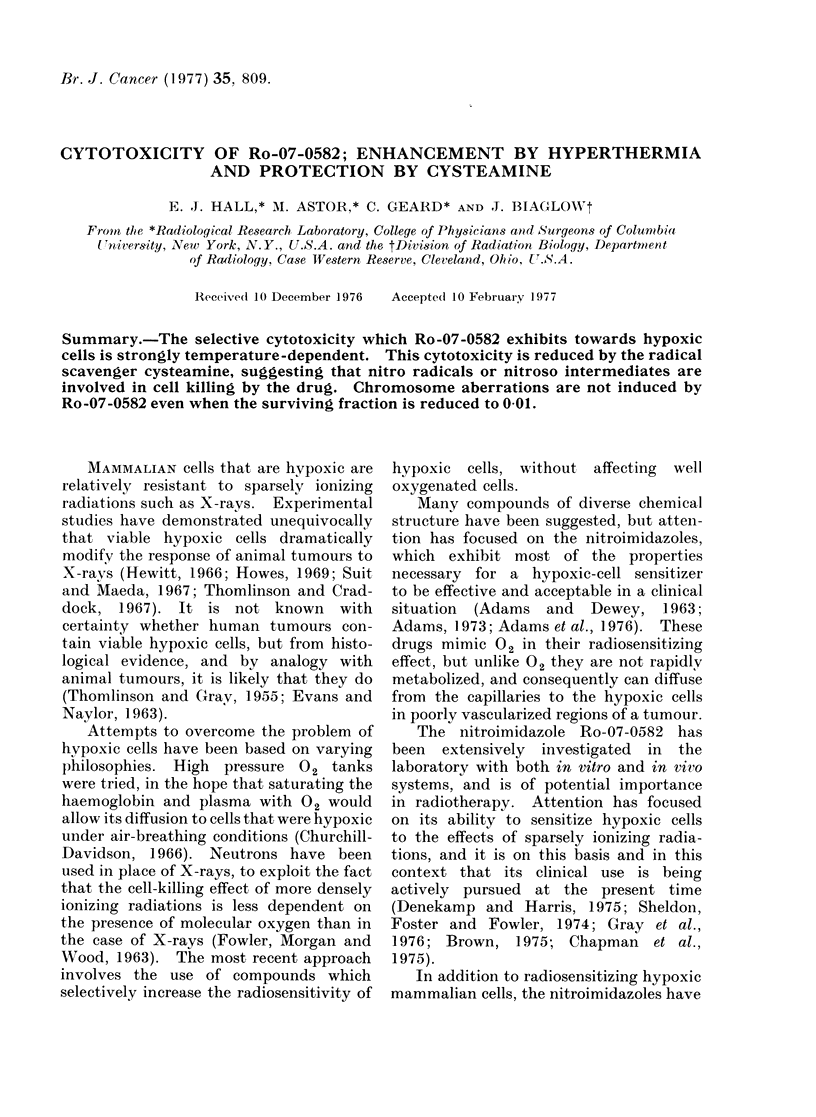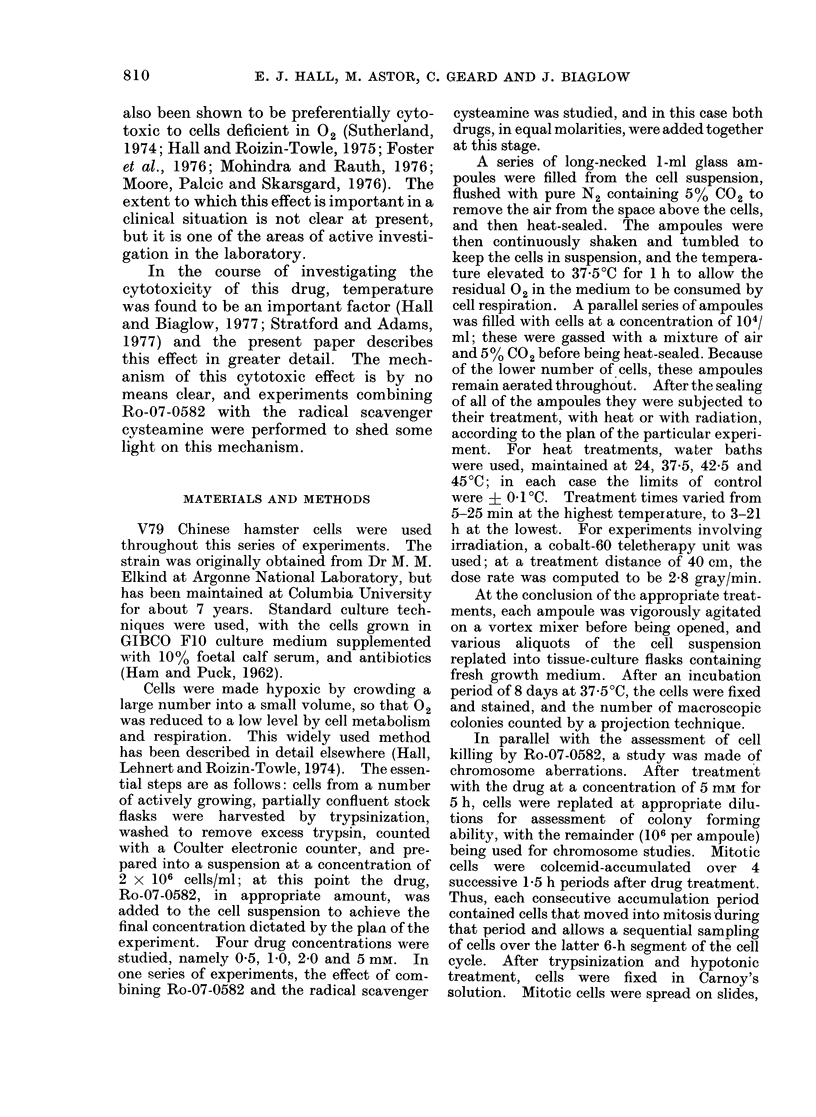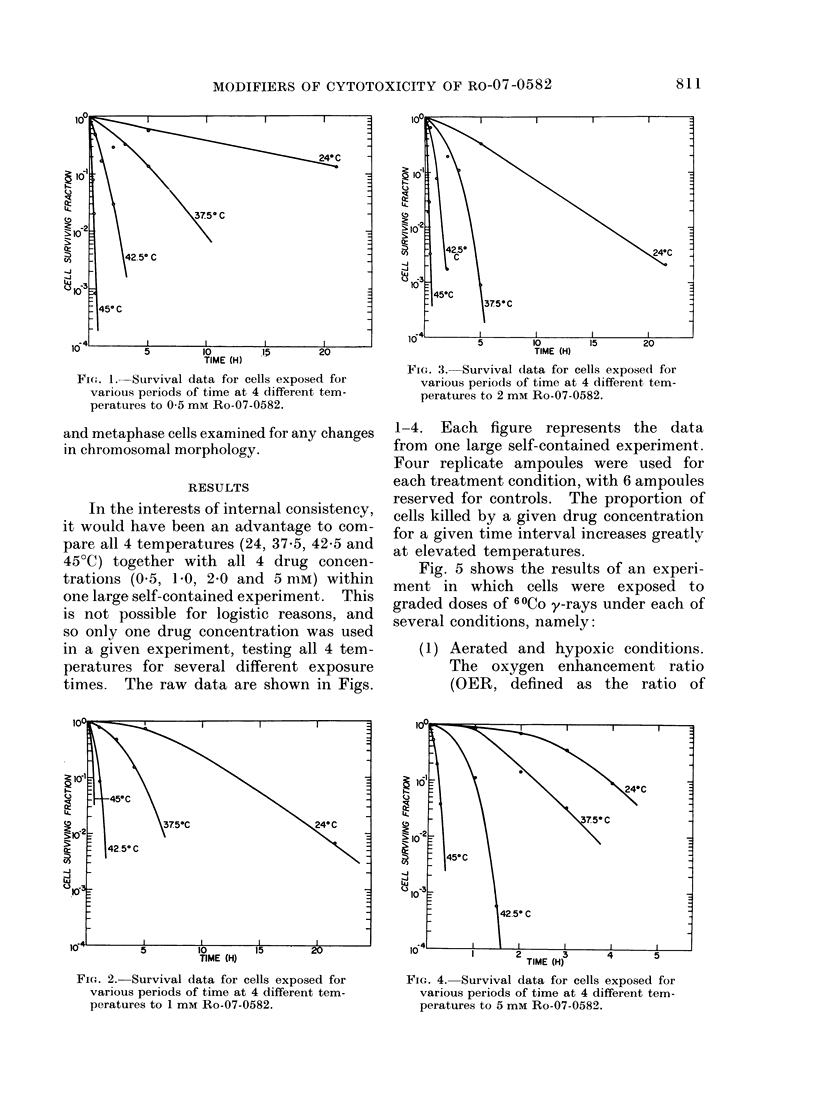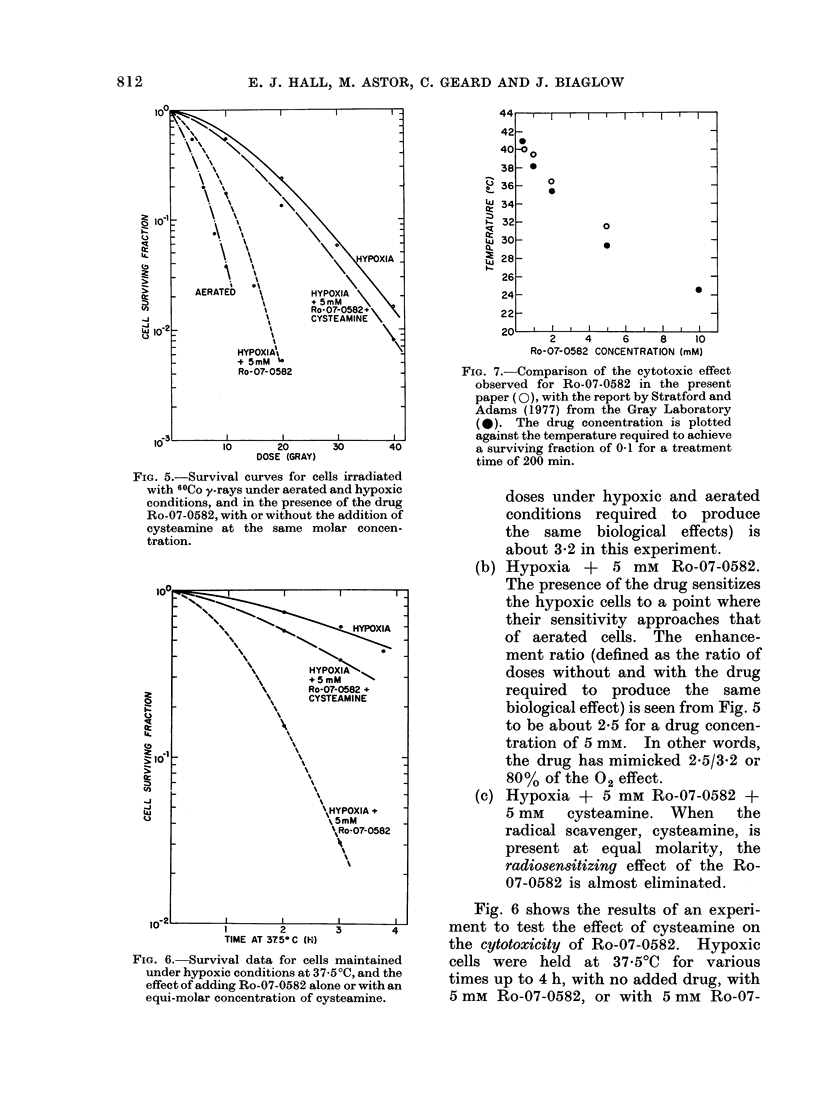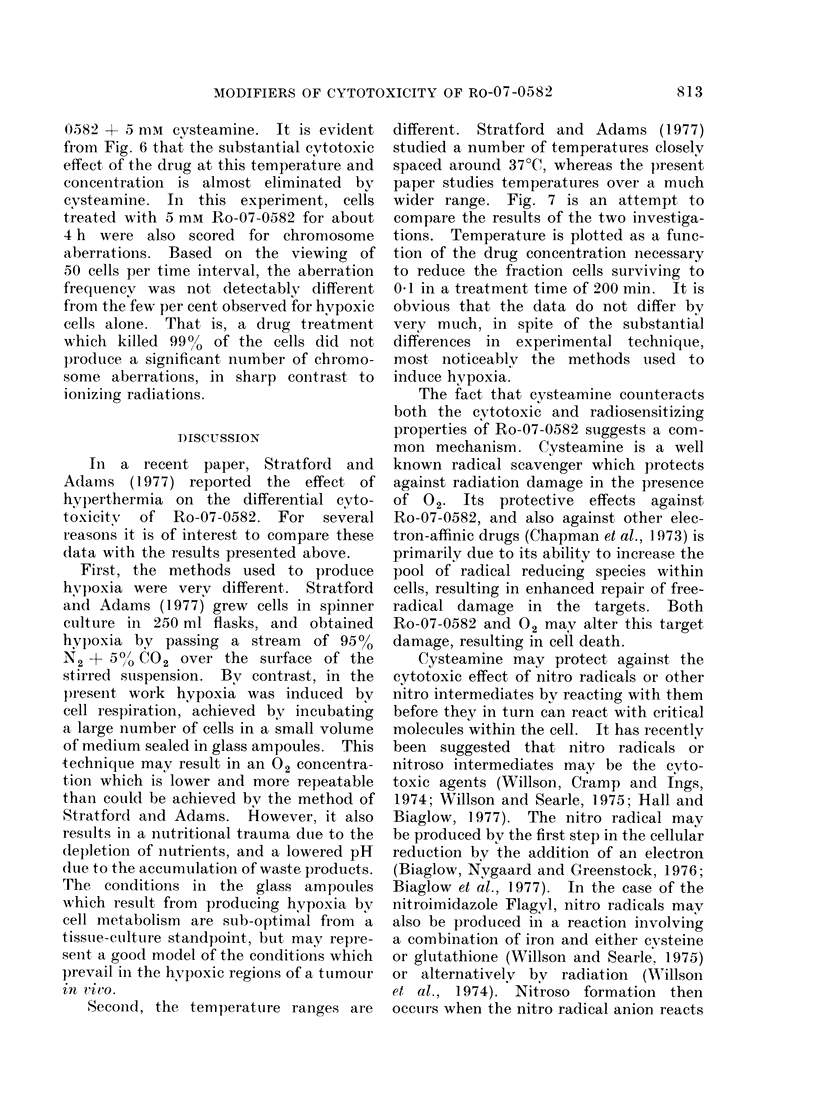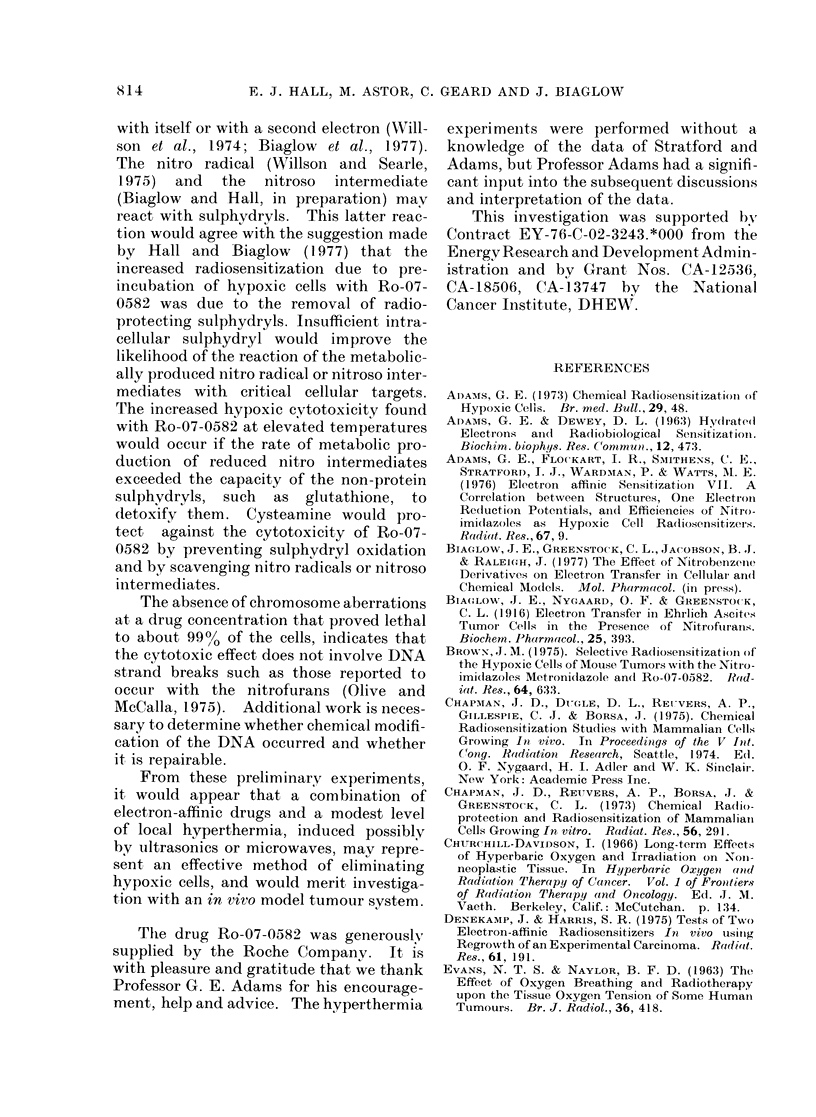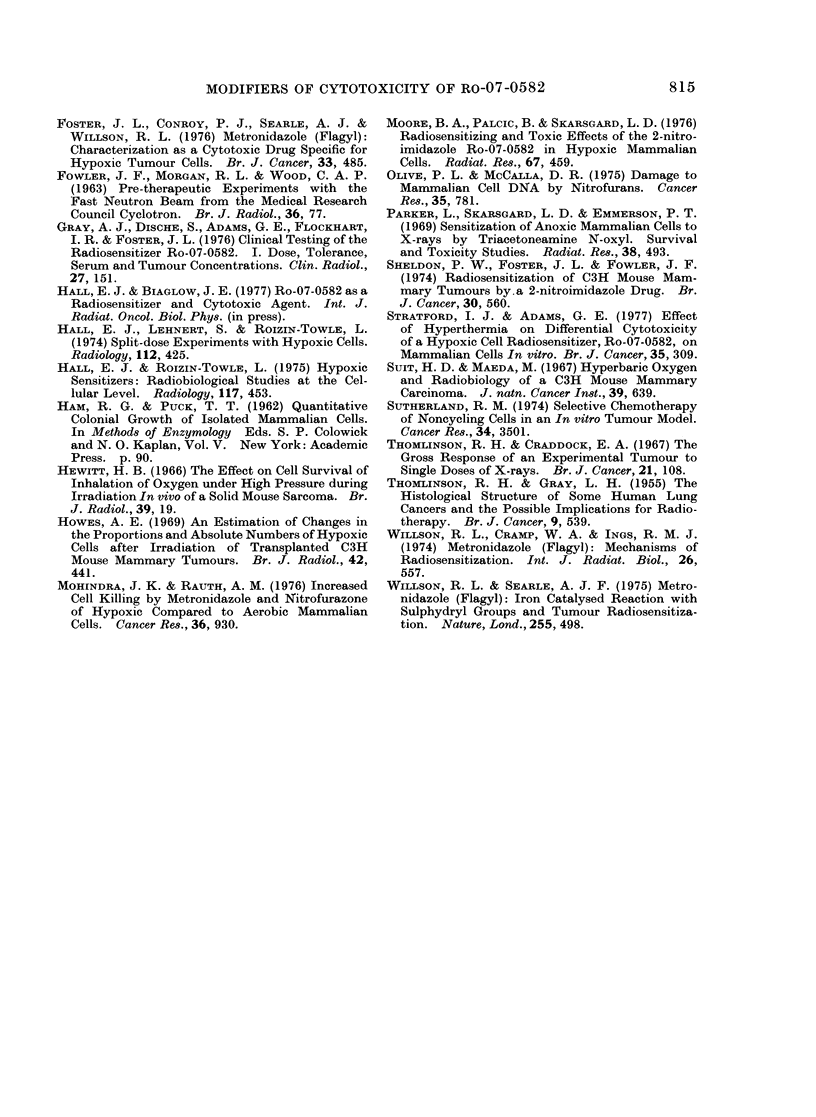# Cytotoxicity of Ro-07-0582; enhancement by hyperthermia and protection by cysteamine.

**DOI:** 10.1038/bjc.1977.122

**Published:** 1977-06

**Authors:** E. J. Hall, M. Astor, C. Geard, J. Biaglow

## Abstract

The selective cytotoxicity which Ro-07-0582 exhibits towards hypoxic cells is strongly temperature-dependent. This cytotoxicity is reduced by the radical scavenger cysteamine, suggesting that nitro radicals or nitroso intermediates are involved in cell killing by the drug. Chromosome aberrations are not induced by Ro-07-0582 even when the surviving fraction is reduced to 0-01.


*Br. J. Cancer* (1977) **35**, 809.

### CYTOTOXICITY OF Ro-07-0582; ENHANCEMENT BY HYPERTHERMIA AND PROTECTION BY CYSTEAMINE

E. J. HALL,* M. ASTOR,* C. GEARD* AND J. BIAGLOW†

*From the *Radiological Research Laboratory, College of Physicians and Surgeons of Columbia University, New York, N.Y., U.S.A. and the †Division of Radiation Biology, Department of Radiology, Case Western Reserve, Cleveland, Ohio, U.S.A.*

Received 10 December 1976 Accepted 10 February 1977

**Summary.—The selective cytotoxicity which Ro-07-0582 exhibits towards hypoxic cells is strongly temperature-dependent. This cytotoxicity is reduced by the radical scavenger cysteamine, suggesting that nitro radicals or nitroso intermediates are involved in cell killing by the drug. Chromosome aberrations are not induced by Ro-07-0582 even when the surviving fraction is reduced to 0·01.**

MAMMALIAN cells that are hypoxic are relatively resistant to sparsely ionizing radiations such as X-rays. Experimental studies have demonstrated unequivocally that viable hypoxic cells dramatically modify the response of animal tumours to X-rays (Hewitt, 1966; Howes, 1969; Suit and Maeda, 1967; Thomlinson and Craddock, 1967). It is not known with certainty whether human tumours contain viable hypoxic cells, but from histological evidence, and by analogy with animal tumours, it is likely that they do (Thomlinson and Gray, 1955; Evans and Naylor, 1963).

Attempts to overcome the problem of hypoxic cells have been based on varying philosophies. High pressure $O_2$ tanks were tried, in the hope that saturating the haemoglobin and plasma with $O_2$ would allow its diffusion to cells that were hypoxic under air-breathing conditions (Churchill-Davidson, 1966). Neutrons have been used in place of X-rays, to exploit the fact that the cell-killing effect of more densely ionizing radiations is less dependent on the presence of molecular oxygen than in the case of X-rays (Fowler, Morgan and Wood, 1963). The most recent approach involves the use of compounds which selectively increase the radiosensitivity of hypoxic cells, without affecting well oxygenated cells.

Many compounds of diverse chemical structure have been suggested, but attention has focused on the nitroimidazoles, which exhibit most of the properties necessary for a hypoxic-cell sensitizer to be effective and acceptable in a clinical situation (Adams and Dewey, 1963; Adams, 1973; Adams *et al.*, 1976). These drugs mimic $O_2$ in their radiosensitizing effect, but unlike $O_2$ they are not rapidly metabolized, and consequently can diffuse from the capillaries to the hypoxic cells in poorly vascularized regions of a tumour.

The nitroimidazole Ro-07-0582 has been extensively investigated in the laboratory with both *in vitro* and *in vivo* systems, and is of potential importance in radiotherapy. Attention has focused on its ability to sensitize hypoxic cells to the effects of sparsely ionizing radiations, and it is on this basis and in this context that its clinical use is being actively pursued at the present time (Denekamp and Harris, 1975; Sheldon, Foster and Fowler, 1974; Gray *et al.*, 1976; Brown, 1975; Chapman *et al.*, 1975).

In addition to radiosensitizing hypoxic mammalian cells, the nitroimidazoles have also been shown to be preferentially cytotoxic to cells deficient in $O_2$ (Sutherland, 1974; Hall and Roizin-Towle, 1975; Foster *et al.*, 1976; Mohindra and Rauth, 1976; Moore, Palcic and Skarsgard, 1976). The extent to which this effect is important in a clinical situation is not clear at present, but it is one of the areas of active investigation in the laboratory.

In the course of investigating the cytotoxicity of this drug, temperature was found to be an important factor (Hall and Biaglow, 1977; Stratford and Adams, 1977) and the present paper describes this effect in greater detail. The mechanism of this cytotoxic effect is by no means clear, and experiments combining Ro-07-0582 with the radical scavenger cysteamine were performed to shed some light on this mechanism.

### MATERIALS AND METHODS

V79 Chinese hamster cells were used throughout this series of experiments. The strain was originally obtained from Dr M. M. Elkind at Argonne National Laboratory, but has been maintained at Columbia University for about 7 years. Standard culture techniques were used, with the cells grown in GIBCO F10 culture medium supplemented with 10% foetal calf serum, and antibiotics (Ham and Puck, 1962).

Cells were made hypoxic by crowding a large number into a small volume, so that $O_2$ was reduced to a low level by cell metabolism and respiration. This widely used method has been described in detail elsewhere (Hall, Lehnert and Roizin-Towle, 1974). The essential steps are as follows: cells from a number of actively growing, partially confluent stock flasks were harvested by trypsinization, washed to remove excess trypsin, counted with a Coulter electronic counter, and prepared into a suspension at a concentration of $2 \times 10^6$ cells/ml; at this point the drug, Ro-07-0582, in appropriate amount, was added to the cell suspension to achieve the final concentration dictated by the plan of the experiment. Four drug concentrations were studied, namely 0·5, 1·0, 2·0 and 5 mM. In one series of experiments, the effect of combining Ro-07-0582 and the radical scavenger cysteamine was studied, and in this case both drugs, in equal molarities, were added together at this stage.

A series of long-necked 1-ml glass ampoules were filled from the cell suspension, flushed with pure $N_2$ containing 5% $CO_2$ to remove the air from the space above the cells, and then heat-sealed. The ampoules were then continuously shaken and tumbled to keep the cells in suspension, and the temperature elevated to 37·5°C for 1 h to allow the residual $O_2$ in the medium to be consumed by cell respiration. A parallel series of ampoules was filled with cells at a concentration of $10^4$/ml; these were gassed with a mixture of air and 5% $CO_2$ before being heat-sealed. Because of the lower number of cells, these ampoules remain aerated throughout. After the sealing of all of the ampoules they were subjected to their treatment, with heat or with radiation, according to the plan of the particular experiment. For heat treatments, water baths were used, maintained at 24, 37·5, 42·5 and 45°C; in each case the limits of control were $\pm$ 0·1°C. Treatment times varied from 5–25 min at the highest temperature, to 3–21 h at the lowest. For experiments involving irradiation, a cobalt-60 teletherapy unit was used; at a treatment distance of 40 cm, the dose rate was computed to be 2·8 gray/min.

At the conclusion of the appropriate treatments, each ampoule was vigorously agitated on a vortex mixer before being opened, and various aliquots of the cell suspension replated into tissue-culture flasks containing fresh growth medium. After an incubation period of 8 days at 37·5°C, the cells were fixed and stained, and the number of macroscopic colonies counted by a projection technique.

In parallel with the assessment of cell killing by Ro-07-0582, a study was made of chromosome aberrations. After treatment with the drug at a concentration of 5 mM for 5 h, cells were replated at appropriate dilutions for assessment of colony forming ability, with the remainder ($10^6$ per ampoule) being used for chromosome studies. Mitotic cells were colcemid-accumulated over 4 successive 1·5 h periods after drug treatment. Thus, each consecutive accumulation period contained cells that moved into mitosis during that period and allows a sequential sampling of cells over the latter 6-h segment of the cell cycle. After trypsinization and hypotonic treatment, cells were fixed in Carnoy's solution. Mitotic cells were spread on slides,

FIG. 1.—Survival data for cells exposed for various periods of time at 4 different temperatures to 0·5 mM Ro-07-0582.

FIG. 3.—Survival data for cells exposed for various periods of time at 4 different temperatures to 2 mM Ro-07-0582.

and metaphase cells examined for any changes in chromosomal morphology.

### RESULTS

In the interests of internal consistency, it would have been an advantage to compare all 4 temperatures (24, 37·5, 42·5 and 45°C) together with all 4 drug concentrations (0·5, 1·0, 2·0 and 5 mM) within one large self-contained experiment. This is not possible for logistic reasons, and so only one drug concentration was used in a given experiment, testing all 4 temperatures for several different exposure times. The raw data are shown in Figs. 1–4. Each figure represents the data from one large self-contained experiment. Four replicate ampoules were used for each treatment condition, with 6 ampoules reserved for controls. The proportion of cells killed by a given drug concentration for a given time interval increases greatly at elevated temperatures.

Fig. 5 shows the results of an experiment in which cells were exposed to graded doses of $^{60}Co$ $\gamma$-rays under each of several conditions, namely:

(1) Aerated and hypoxic conditions. The oxygen enhancement ratio (OER, defined as the ratio of

FIG. 2.—Survival data for cells exposed for various periods of time at 4 different temperatures to 1 mM Ro-07-0582.

FIG. 4.—Survival data for cells exposed for various periods of time at 4 different temperatures to 5 mM Ro-07-0582.

Fig. 5.—Survival curves for cells irradiated with $^{60}$Co $\gamma$-rays under aerated and hypoxic conditions, and in the presence of the drug Ro-07-0582, with or without the addition of cysteamine at the same molar concentration.

Fig. 7.—Comparison of the cytotoxic effect observed for Ro-07-0582 in the present paper (○), with the report by Stratford and Adams (1977) from the Gray Laboratory (●). The drug concentration is plotted against the temperature required to achieve a surviving fraction of 0·1 for a treatment time of 200 min.

Fig. 6.—Survival data for cells maintained under hypoxic conditions at 37·5°C, and the effect of adding Ro-07-0582 alone or with an equi-molar concentration of cysteamine.

doses under hypoxic and aerated conditions required to produce the same biological effects) is about 3·2 in this experiment.

(b) Hypoxia + 5 mM Ro-07-0582. The presence of the drug sensitizes the hypoxic cells to a point where their sensitivity approaches that of aerated cells. The enhancement ratio (defined as the ratio of doses without and with the drug required to produce the same biological effect) is seen from Fig. 5 to be about 2·5 for a drug concentration of 5 mM. In other words, the drug has mimicked 2·5/3·2 or 80% of the $O_2$ effect.

(c) Hypoxia + 5 mM Ro-07-0582 + 5 mM cysteamine. When the radical scavenger, cysteamine, is present at equal molarity, the *radiosensitizing* effect of the Ro-07-0582 is almost eliminated.

Fig. 6 shows the results of an experiment to test the effect of cysteamine on the *cytotoxicity* of Ro-07-0582. Hypoxic cells were held at 37·5°C for various times up to 4 h, with no added drug, with 5 mM Ro-07-0582, or with 5 mM Ro-07-

0582 + 5 mM cysteamine. It is evident from Fig. 6 that the substantial cytotoxic effect of the drug at this temperature and concentration is almost eliminated by cysteamine. In this experiment, cells treated with 5 mM Ro-07-0582 for about 4 h were also scored for chromosome aberrations. Based on the viewing of 50 cells per time interval, the aberration frequency was not detectably different from the few per cent observed for hypoxic cells alone. That is, a drug treatment which killed 99% of the cells did not produce a significant number of chromosome aberrations, in sharp contrast to ionizing radiations.

### DISCUSSION

In a recent paper, Stratford and Adams (1977) reported the effect of hyperthermia on the differential cytotoxicity of Ro-07-0582. For several reasons it is of interest to compare these data with the results presented above.

First, the methods used to produce hypoxia were very different. Stratford and Adams (1977) grew cells in spinner culture in 250 ml flasks, and obtained hypoxia by passing a stream of 95% $N_2$ + 5% $CO_2$ over the surface of the stirred suspension. By contrast, in the present work hypoxia was induced by cell respiration, achieved by incubating a large number of cells in a small volume of medium sealed in glass ampoules. This technique may result in an $O_2$ concentration which is lower and more repeatable than could be achieved by the method of Stratford and Adams. However, it also results in a nutritional trauma due to the depletion of nutrients, and a lowered pH due to the accumulation of waste products. The conditions in the glass ampoules which result from producing hypoxia by cell metabolism are sub-optimal from a tissue-culture standpoint, but may represent a good model of the conditions which prevail in the hypoxic regions of a tumour *in vivo*.

Second, the temperature ranges are different. Stratford and Adams (1977) studied a number of temperatures closely spaced around 37°C, whereas the present paper studies temperatures over a much wider range. Fig. 7 is an attempt to compare the results of the two investigations. Temperature is plotted as a function of the drug concentration necessary to reduce the fraction cells surviving to 0·1 in a treatment time of 200 min. It is obvious that the data do not differ by very much, in spite of the substantial differences in experimental technique, most noticeably the methods used to induce hypoxia.

The fact that cysteamine counteracts both the cytotoxic and radiosensitizing properties of Ro-07-0582 suggests a common mechanism. Cysteamine is a well known radical scavenger which protects against radiation damage in the presence of $O_2$. Its protective effects against Ro-07-0582, and also against other electron-affinic drugs (Chapman *et al.*, 1973) is primarily due to its ability to increase the pool of radical reducing species within cells, resulting in enhanced repair of free-radical damage in the targets. Both Ro-07-0582 and $O_2$ may alter this target damage, resulting in cell death.

Cysteamine may protect against the cytotoxic effect of nitro radicals or other nitro intermediates by reacting with them before they in turn can react with critical molecules within the cell. It has recently been suggested that nitro radicals or nitroso intermediates may be the cytotoxic agents (Willson, Cramp and Ings, 1974; Willson and Searle, 1975; Hall and Biaglow, 1977). The nitro radical may be produced by the first step in the cellular reduction by the addition of an electron (Biaglow, Nygaard and Greenstock, 1976; Biaglow *et al.*, 1977). In the case of the nitroimidazole Flagyl, nitro radicals may also be produced in a reaction involving a combination of iron and either cysteine or glutathione (Willson and Searle, 1975) or alternatively by radiation (Willson *et al.*, 1974). Nitroso formation then occurs when the nitro radical anion reacts with itself or with a second electron (Willson *et al.*, 1974; Biaglow *et al.*, 1977). The nitro radical (Willson and Searle, 1975) and the nitroso intermediate (Biaglow and Hall, in preparation) may react with sulphydryls. This latter reaction would agree with the suggestion made by Hall and Biaglow (1977) that the increased radiosensitization due to preincubation of hypoxic cells with Ro-07-0582 was due to the removal of radioprotecting sulphydryls. Insufficient intracellular sulphydryl would improve the likelihood of the reaction of the metabolically produced nitro radical or nitroso intermediates with critical cellular targets. The increased hypoxic cytotoxicity found with Ro-07-0582 at elevated temperatures would occur if the rate of metabolic production of reduced nitro intermediates exceeded the capacity of the non-protein sulphydryls, such as glutathione, to detoxify them. Cysteamine would protect against the cytotoxicity of Ro-07-0582 by preventing sulphydryl oxidation and by scavenging nitro radicals or nitroso intermediates.

The absence of chromosome aberrations at a drug concentration that proved lethal to about 99% of the cells, indicates that the cytotoxic effect does not involve DNA strand breaks such as those reported to occur with the nitrofurans (Olive and McCalla, 1975). Additional work is necessary to determine whether chemical modification of the DNA occurred and whether it is repairable.

From these preliminary experiments, it would appear that a combination of electron-affinic drugs and a modest level of local hyperthermia, induced possibly by ultrasonics or microwaves, may represent an effective method of eliminating hypoxic cells, and would merit investigation with an *in vivo* model tumour system.

The drug Ro-07-0582 was generously supplied by the Roche Company. It is with pleasure and gratitude that we thank Professor G. E. Adams for his encouragement, help and advice. The hyperthermia experiments were performed without a knowledge of the data of Stratford and Adams, but Professor Adams had a significant input into the subsequent discussions and interpretation of the data.

This investigation was supported by Contract EY-76-C-02-3243.*000 from the Energy Research and Development Administration and by Grant Nos. CA-12536, CA-18506, CA-13747 by the National Cancer Institute, DHEW.

### REFERENCES

ADAMS, G. E. (1973) Chemical Radiosensitization of Hypoxic Cells. *Br. med. Bull.*, **29**, 48.

ADAMS, G. E. & DEWEY, D. L. (1963) Hydrated Electrons and Radiobiological Sensitization. *Biochim. biophys. Res. Commun.*, **12**, 473.

ADAMS, G. E., FLOCKART, I. R., SMITHENS, C. E., STRATFORD, I. J., WARDMAN, P. & WATTS, M. E. (1976) Electron affinic Sensitization VII. A Correlation between Structures, One Electron Reduction Potentials, and Efficiencies of Nitroimidazoles as Hypoxic Cell Radiosensitizers. *Radiat. Res.*, **67**, 9.

BIAGLOW, J. E., GREENSTOCK, C. L., JACOBSON, B. J. & RALEIGH, J. (1977) The Effect of Nitrobenzene Derivatives on Electron Transfer in Cellular and Chemical Models. *Mol. Pharmacol.* (in press).

BIAGLOW, J. E., NYGAARD, O. F. & GREENSTOCK, C. L. (1916) Electron Transfer in Ehrlich Ascites Tumor Cells in the Presence of Nitrofurans. *Biochem. Pharmacol.*, **25**, 393.

BROWN, J. M. (1975). Selective Radiosensitization of the Hypoxic Cells of Mouse Tumors with the Nitroimidazoles Metronidazole and Ro-07-0582. *Radiat. Res.*, **64**, 633.

CHAPMAN, J. D., DUGLE, D. L., REUVERS, A. P., GILLESPIE, C. J. & BORSA, J. (1975). Chemical Radiosensitization Studies with Mammalian Cells Growing *In vivo*. In *Proceedings of the V Int. Cong. Radiation Research*, Seattle, 1974. Ed. O. F. Nygaard, H. I. Adler and W. K. Sinclair. New York: Academic Press Inc.

CHAPMAN, J. D., REUVERS, A. P., BORSA, J. & GREENSTOCK, C. L. (1973) Chemical Radioprotection and Radiosensitization of Mammalian Cells Growing *In vitro*. *Radiat. Res.*, **56**, 291.

CHURCHILL-DAVIDSON, I. (1966) Long-term Effects of Hyperbaric Oxygen and Irradiation on Non-neoplastic Tissue. In *Hyperbaric Oxygen and Radiation Therapy of Cancer. Vol. 1 of Frontiers of Radiation Therapy and Oncology*. Ed. J. M. Vaeth. Berkeley, Calif.: McCutchan. p. 134.

DENEKAMP, J. & HARRIS, S. R. (1975) Tests of Two Electron-affinic Radiosensitizers *In vivo* using Regrowth of an Experimental Carcinoma. *Radiat. Res.*, **61**, 191.

EVANS, N. T. S. & NAYLOR, B. F. D. (1963) The Effect of Oxygen Breathing and Radiotherapy upon the Tissue Oxygen Tension of Some Human Tumours. *Br. J. Radiol.*, **36**, 418.

FOSTER, J. L., CONROY, P. J., SEARLE, A. J. & WILLSON, R. L. (1976) Metronidazole (Flagyl): Characterization as a Cytotoxic Drug Specific for Hypoxic Tumour Cells. *Br. J. Cancer*, **33**, 485.

FOWLER, J. F., MORGAN, R. L. & WOOD, C. A. P. (1963) Pre-therapeutic Experiments with the Fast Neutron Beam from the Medical Research Council Cyclotron. *Br. J. Radiol.*, **36**, 77.

GRAY, A. J., DISCHE, S., ADAMS, G. E., FLOCKHART, I. R. & FOSTER, J. L. (1976) Clinical Testing of the Radiosensitizer Ro-07-0582. I. Dose, Tolerance, Serum and Tumour Concentrations. *Clin. Radiol.*, **27**, 151.

HALL, E. J. & BIAGLOW, J. E. (1977) Ro-07-0582 as a Radiosensitizer and Cytotoxic Agent. *Int. J. Radiat. Oncol. Biol. Phys.* (in press).

HALL, E. J., LEHNERT, S. & ROIZIN-TOWLE, L. (1974) Split-dose Experiments with Hypoxic Cells. *Radiology*, **112**, 425.

HALL, E. J. & ROIZIN-TOWLE, L. (1975) Hypoxic Sensitizers: Radiobiological Studies at the Cellular Level. *Radiology*, **117**, 453.

HAM, R. G. & PUCK, T. T. (1962) Quantitative Colonial Growth of Isolated Mammalian Cells. In *Methods of Enzymology* Eds. S. P. Colowick and N. O. Kaplan, Vol. V. New York: Academic Press. p. 90.

HEWITT, H. B. (1966) The Effect on Cell Survival of Inhalation of Oxygen under High Pressure during Irradiation *In vivo* of a Solid Mouse Sarcoma. *Br. J. Radiol.*, **39**, 19.

HOWES, A. E. (1969) An Estimation of Changes in the Proportions and Absolute Numbers of Hypoxic Cells after Irradiation of Transplanted C3H Mouse Mammary Tumours. *Br. J. Radiol.*, **42**, 441.

MOHINDRA, J. K. & RAUTH, A. M. (1976) Increased Cell Killing by Metronidazole and Nitrofurazone of Hypoxic Compared to Aerobic Mammalian Cells. *Cancer Res.*, **36**, 930.

MOORE, B. A., PALCIC, B. & SKARSGARD, L. D. (1976) Radiosensitizing and Toxic Effects of the 2-nitroimidazole Ro-07-0582 in Hypoxic Mammalian Cells. *Radiat. Res.*, **67**, 459.

OLIVE, P. L. & MCCALLA, D. R. (1975) Damage to Mammalian Cell DNA by Nitrofurans. *Cancer Res.*, **35**, 781.

PARKER, L., SKARSGARD, L. D. & EMMERSON, P. T. (1969) Sensitization of Anoxic Mammalian Cells to X-rays by Triacetoneamine N-oxyl. Survival and Toxicity Studies. *Radiat. Res.*, **38**, 493.

SHELDON, P. W., FOSTER, J. L. & FOWLER, J. F. (1974) Radiosensitization of C3H Mouse Mammary Tumours by a 2-nitroimidazole Drug. *Br. J. Cancer*, **30**, 560.

STRATFORD, I. J. & ADAMS, G. E. (1977) Effect of Hyperthermia on Differential Cytotoxicity of a Hypoxic Cell Radiosensitizer, Ro-07-0582, on Mammalian Cells *In vitro*. *Br. J. Cancer*, **35**, 309.

SUIT, H. D. & MAEDA, M. (1967) Hyperbaric Oxygen and Radiobiology of a C3H Mouse Mammary Carcinoma. *J. natn. Cancer Inst.*, **39**, 639.

SUTHERLAND, R. M. (1974) Selective Chemotherapy of Noncycling Cells in an *In vitro* Tumour Model. *Cancer Res.*, **34**, 3501.

THOMLINSON, R. H. & CRADDOCK, E. A. (1967) The Gross Response of an Experimental Tumour to Single Doses of X-rays. *Br. J. Cancer*, **21**, 108.

THOMLINSON, R. H. & GRAY, L. H. (1955) The Histological Structure of Some Human Lung Cancers and the Possible Implications for Radiotherapy. *Br. J. Cancer*, **9**, 539.

WILLSON, R. L., CRAMP, W. A. & INGS, R. M. J. (1974) Metronidazole (Flagyl): Mechanisms of Radiosensitization. *Int. J. Radiat. Biol.*, **26**, 557.

WILLSON, R. L. & SEARLE, A. J. F. (1975) Metronidazole (Flagyl): Iron Catalysed Reaction with Sulphydryl Groups and Tumour Radiosensitization. *Nature, Lond.*, **255**, 498.